# Automated scratching detection system for black mouse using deep learning

**DOI:** 10.3389/fphys.2022.939281

**Published:** 2022-07-22

**Authors:** Naoaki Sakamoto, Taiga Haraguchi, Koji Kobayashi, Yusuke Miyazaki, Takahisa Murata

**Affiliations:** Department of Animal Radiology, Graduate School of Agricultural and Life Sciences, The University of Tokyo, Tokyo, Japan

**Keywords:** scratching behavior, itching, neural network, convolutional neural network, pruritus

## Abstract

The evaluation of scratching behavior is important in experimental animals because there is significant interest in elucidating mechanisms and developing medications for itching. The scratching behavior is classically quantified by human observation, but it is labor-intensive and has low throughput. We previously established an automated scratching detection method using a convolutional recurrent neural network (CRNN). The established CRNN model was trained by white mice (BALB/c), and it could predict their scratching bouts and duration. However, its performance in black mice (C57BL/6) is insufficient. Here, we established a model for black mice to increase prediction accuracy. Scratching behavior in black mice was elicited by serotonin administration, and their behavior was recorded using a video camera. The videos were carefully observed, and each frame was manually labeled as scratching or other behavior. The CRNN model was trained using the labels and predicted the first-look videos. In addition, posterior filters were set to remove unlikely short predictions. The newly trained CRNN could sufficiently detect scratching behavior in black mice (sensitivity, 98.1%; positive predictive rate, 94.0%). Thus, our established CRNN and posterior filter successfully predicted the scratching behavior in black mice, highlighting that our workflow can be useful, regardless of the mouse strain.

## Introduction

Over 70 million people suffer from itching worldwide (Institute for Health Metrics and Evaluation, 2020). Chronic itching is sometimes associated with serious problems, such as sleep impairments and depression, and decreases the patients’ quality of life (Lee et al., 2021). Although various medications, including antihistamine drugs are typically used for treatments, detailed mechanisms of refractory cases remain unknown (Yosipovitch et al., 2018). Therefore, researchers have established various rodent itching models (Hoeck et al., 2016) and have explored the mechanisms and drug candidates.

Since we cannot directly assess itching sensation in rodents, we usually evaluate itching by observing scratching. In most laboratories, visual observation is the standard method for measuring scratching bouts and durations. However, it has low throughput and places physical and mental burdens on researchers. Therefore, automated tools are required to promote research into itching.

Recently, we reported an automated scratching detection method using a convolutional recurrent neural network (CRNN) (Kobayashi et al., 2021). This method can detect scratching behavior in white mice (BALB/c). In addition, only a simple recording environment was required to use our method: a video camera, a cage, and a GPU-mounted computer. These characteristics are superior to existing methods requiring special equipment (Inagaki et al., 2003; Elliott et al., 2017; Tarrasón et al., 2017). However, in a previous study, the established method could not sufficiently predict the scratching behavior in black mice (C57BL/6). Because black mice are also widely used in research, including for itching (Jin et al., 2009), the development of an accurate automated prediction method for black mice, as is the case for white mice, is desired.

This study aimed to improve our workflow and develop an accurate automated prediction method for black mice. The CRNN and posterior filter combination successfully detected scratching behaviors in black mice. Together with previous works, our proposed workflow can accelerate research on itching.

## Materials and methods

### Mice

C57BL/6 mice (12–18 weeks old; males, *n* = 9) were used in this study. All the experiments were approved by the Institutional Animal Care and Use Committee of the University of Tokyo (P19-079). Animal care and treatment were performed in accordance with the guidelines outlined in the Guide to Animal Use and Care of the University of Tokyo.

### Serotonin treatment and video recording

Serotonin (10 ug in 10 ul saline; Serotonin hydrochloride, Sigma Aldrich, St. Louis, MO) was intradermally injected into the backs of C57BL/6 mice to induce scratching behavior. After treatment, the mice were placed into a white square arena (37 cm × 25 cm × 22 cm), and their behavior was recorded for 20–30 min using a video camera (HDR-CX720V, Sony, Tokyo, Japan). The detailed recording conditions were as follows: frame rate, 60 Hz; resolution, 1,920 × 1,080 pixels. We split the recorded videos every about 10.5 min and selected 21 videos in which several scratching bouts were observed.

### Manual labelling of scratching

We defined scratching behavior as the rapid, repetitive, and back-and-forth movement of the hind limb toward the injection site, as previously defined (Kobayashi et al., 2021). The recorded videos were carefully observed and each frame was labeled as “scratching”: 1 or “other behaviors”: 0.

### Image pre-processing

All the recorded videos were divided into frames. The frames were pre-processed as previously described (Kobayashi et al., 2021). Briefly, differential images between successive frames were generated and cropped around the geometric center of the mouse into a square shape. They were converted to gray scale, binarized, and resized to 200 × 200 pixels. The binarization threshold was set to 15. To consider the temporal changes before and after the frame at time t, 21 pre-processed images from t-10 to t+10 were collected. Hereafter, we call them as “segment”. The label of the frame at time t is assigned to the label of the segments at time t.

### Convolutional recurrent neural network

The architecture and hyperparameters were similar to those described previously (Kobayashi et al., 2021). Briefly, the CRNN is composed of three convolution and max pooling layers, two LSTM layers, and five fully connected layers. One hundred segments were randomly selected from those labeled as scratching, and 1,500 segments were randomly selected regardless of labels for every epoch. The images were randomly flipped and rotated by multiples of 20° for data augmentation. An Adam optimizer with a 3 × 10^−5^ learning rate and a binary cross-entropy loss function was used. For the prediction, segments were input into the trained CRNN without data augmentation. The CRNN outputs a decimal value between zero and one for the segments. We defined a segment which value was more than 0.5 as “scratching”.

### Prediction of scratching bouts and duration

A series of continuous predictions were counted as a bout. Duration was calculated as the time period for each bout. When a bout observed by humans was predicted to be several bouts, the duration was predicted as the sum of the time period of predicted bouts.

### Computer hardware and software

Training and prediction of neural networks were conducted on a desktop computer equipped with an Intel Core i9-9900 KS CPU, 64 GB RAM, and NVIDIA GeForce RTX 2080 Ti using the TensorFlow library (version 1.14.0) in Python.

## Results

### Dataset preparation of scratching behavior

Serotonin (10 µg in 10 µL saline) was intradermally injected into the backs of C57BL/6 mice (*n* = 9) to induce scratching behavior. The mice’s behavior was recorded for 20–30 min using a video camera. We split the recorded videos approximately every 10.5 min and obtained 21 videos in which the mice scratched several times. These videos were divided into frames, and each frame was manually labeled as “scratching” or “other behavior.” In addition, the images were pre-processed as previously described (Materials and Methods).

The 21 videos were split into three datasets: 12 videos for the training dataset, four for the validation dataset, and five for the test dataset. Five mice were used to create the training and validation datasets, and the other four mice were used to create the test dataset. The training dataset was used to train the neural network, the validation dataset was used to tune the hyperparameters, and the test dataset was used to evaluate the performance of the trained neural network.

### CRNN training

The CRNN model was constructed as previously described (Kobayashi et al., 2021) and trained with the training dataset. The losses, an index of the difference between CRNN prediction and human labels, for the training dataset were surveyed to understand training progress. The losses gradually decreased and reached almost plateaued at 800 epochs (Supplementary Figure S1A), suggesting that the training progress almost converged at 800 epochs. Thus, we stopped training at that point. When a neural network excessively learns the training datasets, the performance to predict the first-look dataset decreases, which are usually called “overfitting.” We checked the performance of the trained CRNN every 200 epochs using the training and validation datasets whose videos were not used for training. The ratio of mispredicted frames to all frames (error rate) was calculated for both datasets (Supplementary Figure S1B). While the error rate gradually decreased as training progressed for the training dataset, the model at 600 epochs showed the lowest error rate (0.26%) for the validation dataset. Therefore, we used this model with 600 epochs.

### Performance validation and filter application

The performance of the trained model was evaluated using the validation dataset. The sensitivity and specificity were 93.5% (2,645 of 2,830) and 99.9% (149,331 of 149,546). The positive predictive and negative predictive values were 92.5% (2,645 of 2,860) and 99.9% (149,331 of 149,516) (Supplementary Table S1). Besides, we also confirmed that the previous model trained with BALB/c dataset exhibited lower performance (sensitivity, 50.7%; positive predictive rate, 99.5%; Supplementary Table S3), consistent with our previous result (Kobayashi et al., 2021).

**FIGURE 1:**
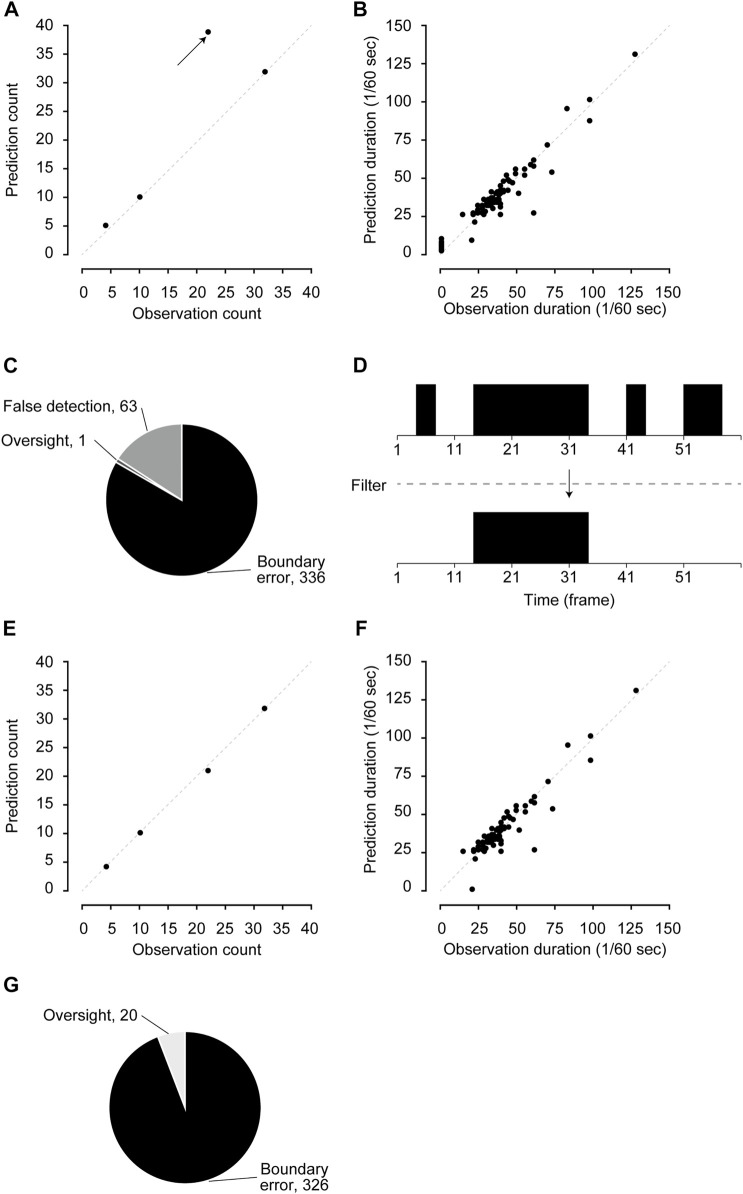
Performance validation and filter application. **(A,B)** The comparison of the number of scratching bouts **(A)** and duration **(B)** between human observation and pre-filtered predictions. **(C)** Details of mis-predicted frames in pre-filtered predictions. **(D)** Schematic figure of the posterior filter. **(E,F)** The comparison of the number of scratching bouts **(E)** and duration **(F)** between human observation and post-filtered predictions. **(G)** Details of mis-predicted frames in post-filtered predictions.

Next, we counted the number of scratching bouts and calculated the duration of each bout. The number of scratching bouts and the duration of each bout predicted by the CRNN were largely comparable to those counted by humans (Figures 1A,B; bouts: *r* = 0.871, duration: *r* = 0.963). However, there was a video in which bouts were overestimated compared to others (indicated by an arrow in Figure 1A).

Mispredicted frames were classified into three categories: 1) boundary errors, 2) false detections, and 3) oversights to investigate the error details. “Boundary errors” were assigned to cases in which the start frame and/or end frame of a series of continuous predictions were shifted compared with those of human labeling. “False detections” were assigned to cases in which the CRNN mislabeled non-scratching behavior as scratching. Oversights were assigned to cases in which the CRNN failed to detect scratching behavior labeled by humans. In the mispredicted frames of the validation dataset, boundary errors were 84.0% (336 of 400), false detections were 15.8% (63 of 400), and oversights were 0.3% (1 of 400) (Figure 1C). In addition, we found that false detections occurred for short periods (one to nine frames) when the mice walked and groomed themselves. Therefore, a posterior filter that removed the predictions for nine or fewer frames was set to improve the predictive performance (Figure 1D).

After the filter was applied, the overestimated bouts were significantly improved, whereas the prediction of durations remained highly correlated with human observations (Figures 1E,F; bouts: *r* = 0.999, duration: *r* = 0.930). In addition, analyzing the details of the errors showed that false detections were completely removed, and oversights remained at low levels (Figures 1C,G). We also confirmed that there were no apparent changes in sensitivity (93.1%), specificity (99.9%), positive predictive rate (94.5%), and negative predictive rate (99.9%, Supplementary Table S2). These results indicate that the posterior filter improves the performance in predicting the number of scratching bouts.

### Performance evaluation using the test dataset

**FIGURE 2:**
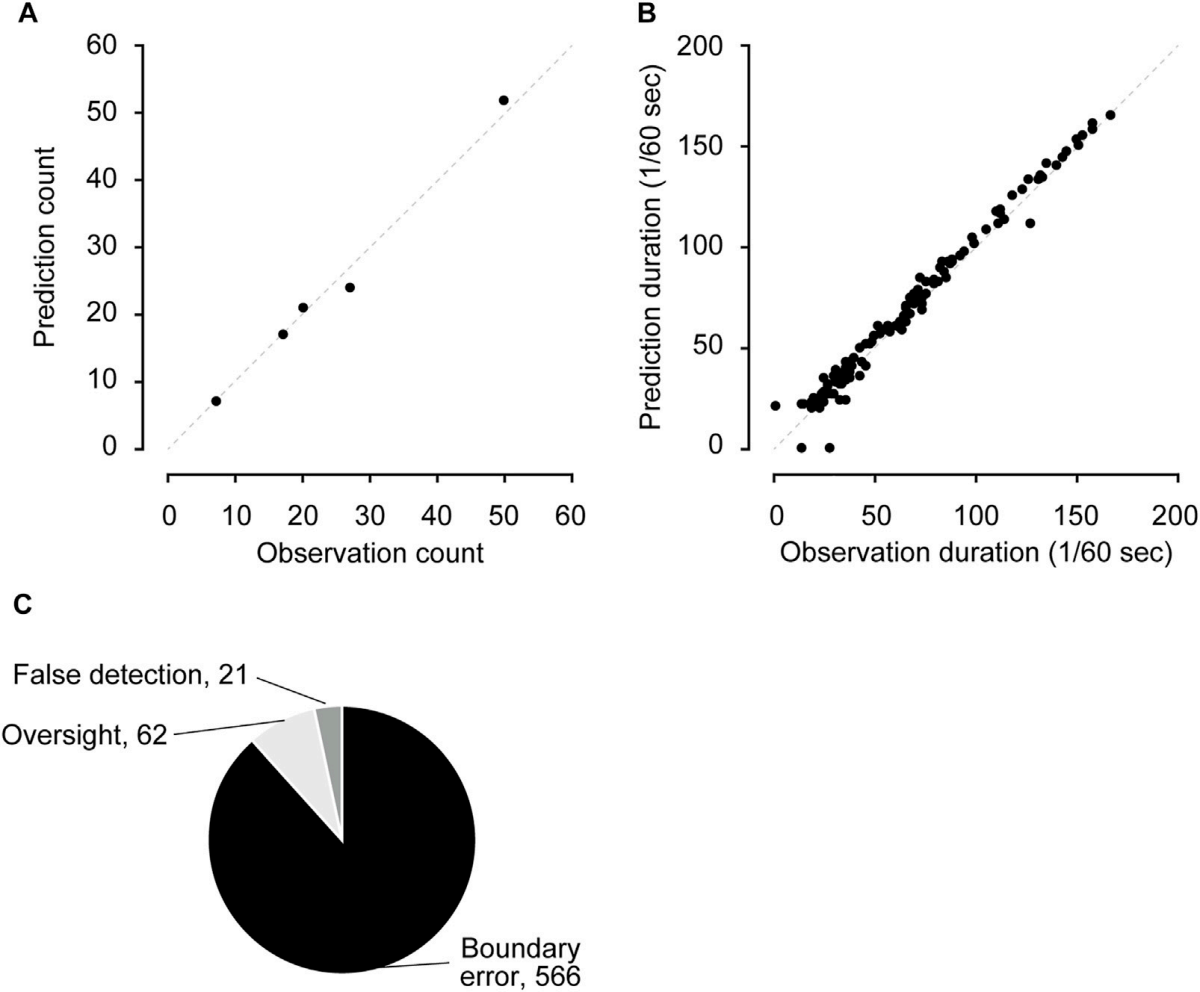
Performance evaluation using the test dataset **(A,B)** The comparison of the number of scratching bouts **(A)** and duration **(B)** between human observation and predictions. **(C)** Details of mis-predicted frames.

We evaluated the trained CRNN and filter performance using the test dataset. The test dataset consisted of five videos not used in the training or validation datasets. The sensitivity and specificity were 98.1% (7,814 of 7,968) and 99.9% (182,082 of 182,577), respectively. The positive predictive and negative predictive values were 94.0% (7,814 in 8,309) and 99.9% (182,082 in 182,236) (Table 1). The number of bouts and duration predicted by the CRNN and filter were highly correlated with those counted by humans (Figures 2A,B; bouts: *r* = 0.994, duration: *r* = 0.991). The most frequent errors were boundary errors (87.2%, 566 in 612); false detections and oversights accounted only for 3.2% (21 of 612) and 9.6% (62 in 612), respectively (Figure 2C). Because boundary errors can occur among human experimenters, these results demonstrate the high performance of the method.

**TABLE 1:** Confusion matrix of post-filtered CRNN prediction for the test dataset.

Post filtered prediction		Predicted label		Sensitivity
		Scratch	Not	
Human observation	Scratch	7,814	154	98.1%
	Not	495	182,082
Positive predictive value		94.0%	

## Discussion

Evaluation of mouse scratching behavior is indispensable for itching research. We previously established a model to automatically detect the scratching behavior of a white BALB/c strain using CRNN (Kobayashi et al., 2021). In this study, we improved the method by introducing a posterior filter and showed that our workflow can be applied to C57BL/6 mice.

Visual assessment of scratching behavior is a general method; however, it is time-consuming and exhausts researchers. Therefore, automated tools have been developed using magnets (Inagaki et al., 2003), sounds (Elliott et al., 2017), vibrations (Tarrasón et al., 2017), and video images (Ishii et al., 2008; Kobayashi et al., 2021). In the present study, we adopted video-based analysis and used simple recording setups: a cage and a video camera. Since this simple setup did not require invasive treatment (Inagaki et al., 2003), our method places less of a burden on experimental animals. In addition, the initial introduction costs into the experimental facilities can be lower, as no special equipment is necessary. Besides, we can easily check predictions by observing recorded video files, whereas it is difficult to make such checks from sounds and vibrations only. Therefore, our method is superior to the existing methods.

Because various mouse strains have been used for itching research (Inagaki et al., 2001; Jin et al., 2009; Hoeck et al., 2016), automated scratching detection methods are desired to be used regardless of strain. However, while humans can easily detect the scratching behavior of any strain, machine learning methods are often affected by differences such as fur colors and sizes. For instance, the neural network model trained using the BALB/c strain dataset did not sufficiently predict the dataset of C57BL/6 strain (Kobayashi et al., 2021). We considered that this failure was caused by slight differences between the training and test datasets and newly created the datasets for C57BL/6 strain. Consequently, the model trained using the new training dataset successfully detected the scratch behavior of C57BL/6 mice. More importantly, we noted that the number of bouts and duration were highly correlated with those of human observations, showing feasibility for practical use. These results suggest that our proposed workflow can operate regardless of the mouse strains, when a specific, tailor-made training dataset is created from scratch.

Machine-learning methods sometimes provide unnatural predictions because they do not consider biological factors. In the previous study, we did not examine whether the predicted periods were sufficiently long for mice to exhibit scratching behavior (Kobayashi et al., 2021). We excluded unlikely short (<10 frames) scratching predictions by applying a posterior filter. This post-processing successfully decreased the number of false detection frames (Figures 1C,G) and improved the prediction of scratching bouts (Figures 1A,E). Because adjusting filters is easier than tuning the hyperparameters of CRNN, the present study showed that the posterior filter could effectively improve the output of machine-learning methods.

However, our workflow has a weakness: the requirement of strain-specific models. Recording videos and manual labeling is laborious work for researchers. For instance, we spent hours labeling 10-min videos in each study (Kobayashi et al., 2021). One possible solution is fine-tuning or transfer-learning, which utilize already trained neural network models. These techniques can reduce the amount of necessary labeling as adopted in DeepLabCut, an animal pose estimation algorithm (Mathis et al., 2018). We are currently investigating the best way to reduce the amount of necessary labeling with fine-tuning or transfer-learning. Another solution is to develop versatile models using a few strains. Although it is beyond the scope of the present study, there is great interest in establishing a model that can be used for any kind of mouse. Creating a mixed dataset for different strains might help neural networks learn the universal features of scratching behavior. Further investigations are required to address these issues.

In conclusion, we established an automated method to detect scratching behavior in C57BL/6 mice using a CRNN. Combined with a previous study, this study showed that our workflow could accelerate itching research.

## Data Availability

The original contributions presented in the study are included in the article/Supplementary Material, further inquiries can be directed to the corresponding author.

## References

[B1] Elliott P. G’Sell M. Snyder L. M. Ross S. E. Ventura V. (2017). Automated acoustic detection of mouse scratching. PLoS One 12, e0179662. 10.1371/journal.pone.0179662 28678797 PMC5497976

[B2] Hoeck E. A. Marker J. B. Gazerani P. Andersen H. H. Arendt-Nielsen L. (2016). Preclinical and human surrogate models of itch. Exp. Dermatol. 25, 750–757. 10.1111/exd.13078 27194117

[B3] Inagaki N. Igeta K. Shiraishi N. Kim J. F. Nagao M. Nakamura N. (2003). Evaluation and characterization of mouse scratching behavior by a new apparatus, MicroAct. Skin. Pharmacol. Appl. Skin. Physiol. 16, 165–175. 10.1159/000069755 12677097

[B4] Inagaki N. Nagao M. Igeta K. Kawasaki H. Kim J. F. Nagai H. (2001). Scratching behavior in various strains of mice. Skin. Pharmacol. Appl. Skin. Physiol. 14, 87–96. 10.1159/000056338 11316967

[B5] Institute for Health Metrics and Evaluation (2020). GBD 2019 cause and risk summary: Pruritus. Seattle, USA: IHME, University of Washington. Accessed 2022/04/29.

[B6] Ishii I. Kurozumi S. Orito K. Matsuda H. (2008). Automatic scratching pattern detection for laboratory mice using high-speed video images. IEEE Trans. Autom. Sci. Eng. 5, 176–182. 10.1109/tase.2007.902868

[B7] Jin H. He R. Oyoshi M. Geha R. S. (2009). Animal models of atopic dermatitis. J. Investig. Dermatol. 129, 31–40. 10.1038/jid.2008.106 19078986 PMC2886143

[B8] Kobayashi K. Matsushita S. Shimizu N. Masuko S. Yamamoto M. Murata T. (2021). Automated detection of mouse scratching behaviour using convolutional recurrent neural network. Sci. Rep. 11, 658. 10.1038/s41598-020-79965-w 33436724 PMC7803777

[B9] Lee J. Suh H. Jung H. Park M. Ahn J. (2021). Association between chronic pruritus, depression, and insomnia: A cross-sectional study. JAAD Int. 3, 54–60. 10.1016/j.jdin.2021.02.004 34409371 PMC8361905

[B10] Mathis A. Mamidanna P. Cury K. M. Abe T. Murthy V. N. Mathis M. W. (2018). DeepLabCut: Markerless pose estimation of user-defined body parts with deep learning. Nat. Neurosci. 21, 1281–1289. 10.1038/s41593-018-0209-y 30127430

[B11] Tarrasón G. Carcasona C. Eichhorn P. Pérez B. Gavaldà A. Godessart N. (2017). Characterization of the chloroquine-induced mouse model of pruritus using an automated behavioural system. Exp. Dermatol. 26, 1105–1111. 10.1111/exd.13392 28605064

[B12] Yosipovitch G. Rosen J. D. Hashimoto T. (2018). Itch: From mechanism to (novel) therapeutic approaches. J. Allergy Clin. Immunol. 142, 1375–1390. 10.1016/j.jaci.2018.09.005 30409247

