# Supplementary material for: Automated scratching detection system for black mouse using deep learning
Source: Front Physiol. 2022 Jul 22;13:939281. doi: 10.3389/fphys.2022.939281 (PMC9352956; doi:10.3389/fphys.2022.939281)
Supplement: Supplementary file 1 [file Table1.DOCX]

Supplementary Material

# Supplementary Figures

**Fig S1**


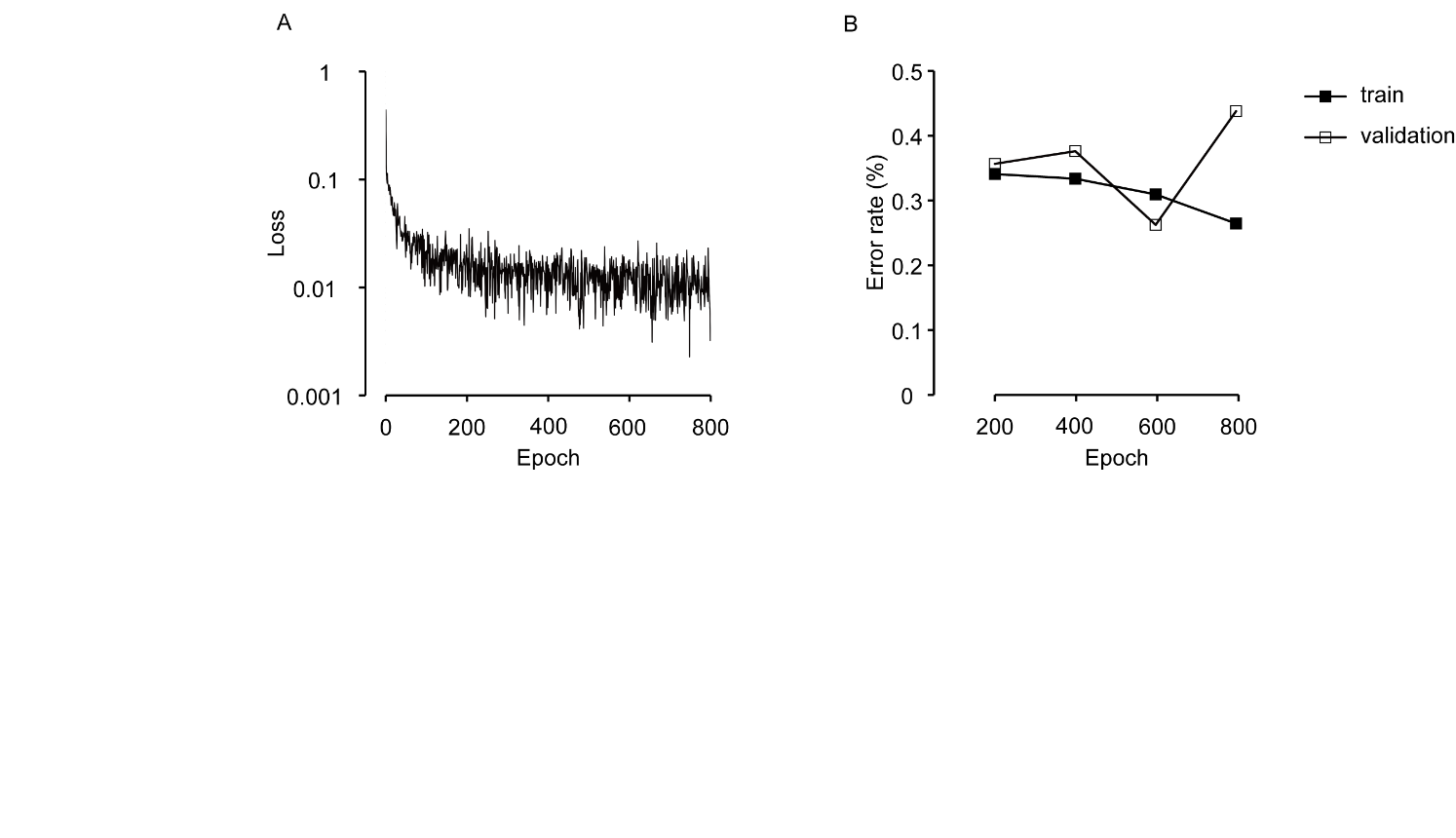


**Fig. S1 Training and validation of CRNN.**

**(A and B)** The transitions of training losses **(A)** and error rates **(B)**

# Supplementary Tables

**Table S1 Confusion matrix of CRNN prediction for the validation dataset.**

| Validation dataset | | Predicted label | | Sensitivity |
| --- | --- | --- | --- | --- |
|  |  | Scratch | Not |  |
| Human observation | Scratch | 2645 | 185 | 93.5% |
|  | Not | 215 | 149331 |  |
| Positive predictive value |  | 92.3% |  |  |

**Table S2 Confusion matrix of post-filtered CRNN prediction for the validation dataset.**

| Validation dataset (filtered) | | Predicted label | | Sensitivity |
| --- | --- | --- | --- | --- |
|  |  | Scratch | Not |  |
| Human observation | Scratch | 2636 | 194 | 93.1% |
|  | Not | 152 | 149394 |  |
| Positive predictive value |  | 94.5% |  |  |

**Table S3. Confusion matrix of the previous CRNN prediction for the validation dataset**

| Validation dataset | | Predicted label | | Sensitivity |
| --- | --- | --- | --- | --- |
|  |  | Scratch | Not |  |
| Human observation | Scratch | 1436 | 1394 | 50.7% |
|  | Not | 7 | 149539 |  |
| Positive predictive value |  | 99.5% |  |  |
